# Simultaneous measurements of 3D wall shear stress and pulse wave velocity in the murine aortic arch

**DOI:** 10.1186/s12968-021-00725-4

**Published:** 2021-03-18

**Authors:** Patrick Winter, Kristina Andelovic, Thomas Kampf, Jan Hansmann, Peter Michael Jakob, Wolfgang Rudolf Bauer, Alma Zernecke, Volker Herold

**Affiliations:** 1 Experimental Physiks V, University of Würzburg, Am Hubland, 97074 Würzburg, Germany; 2 Institute of Experimental Biomedicine, University Hospital Würzburg, Josef-Schneider-Straße 2, 97080 Würzburg, Germany; 3 Medical Clinic and Policlinic I, University Hospital Wuerzburg, Oberdürrbacher Straße 6, 97080 Würzburg, Germany; 4 Department of Diagnostic and Interventional Neuroradiology, University Hospital Würzburg, Josef-Schneider-Straße 11, 97080 Würzburg, Germany; 5 Institute of Electrical Engineering, University of Applied Sciences Würzburg-Schweinfurt (FHWS), Ignaz-Schön-Straße 11, 97421 Schweinfurt, Germany

**Keywords:** 4D flow, Pulse wave velocity, Wall shear stress, Radial, Self-navigation, Mouse, Aortic arch, Atherosclerosis

## Abstract

**Purpose:**

Wall shear stress (WSS) and pulse wave velocity (PWV) are important parameters to characterize blood flow in the vessel wall. Their quantification with flow-sensitive phase-contrast (PC) cardiovascular magnetic resonance (CMR), however, is time-consuming. Furthermore, the measurement of WSS requires high spatial resolution, whereas high temporal resolution is necessary for PWV measurements. For these reasons, PWV and WSS are challenging to measure in one CMR session, making it difficult to directly compare these parameters. By using a retrospective approach with a flexible reconstruction framework, we here aimed to simultaneously assess both PWV and WSS in the murine aortic arch from the same 4D flow measurement.

**Methods:**

Flow was measured in the aortic arch of 18-week-old wildtype (n = 5) and ApoE^−/−^ mice (n = 5) with a self-navigated radial 4D-PC-CMR sequence. Retrospective data analysis was used to reconstruct the same dataset either at low spatial and high temporal resolution (PWV analysis) or high spatial and low temporal resolution (WSS analysis). To assess WSS, the aortic lumen was labeled by semi-automatically segmenting the reconstruction with high spatial resolution. WSS was determined from the spatial velocity gradients at the lumen surface. For calculation of the PWV, segmentation data was interpolated along the temporal dimension. Subsequently, PWV was quantified from the through-plane flow data using the multiple-points transit-time method. Reconstructions with varying frame rates and spatial resolutions were performed to investigate the influence of spatiotemporal resolution on the PWV and WSS quantification.

**Results:**

4D flow measurements were conducted in an acquisition time of only 35 min. Increased peak flow and peak WSS values and lower errors in PWV estimation were observed in the reconstructions with high temporal resolution. Aortic PWV was significantly increased in ApoE^−/−^ mice compared to the control group (1.7 ± 0.2 versus 2.6 ± 0.2 m/s, p < 0.001). Mean WSS magnitude values averaged over the aortic arch were (1.17 ± 0.07) N/m^2^ in wildtype mice and (1.27 ± 0.10) N/m^2^ in ApoE^−/−^ mice.

**Conclusion:**

The post processing algorithm using the flexible reconstruction framework developed in this study permitted quantification of global PWV and 3D-WSS in a single acquisition. The possibility to assess both parameters in only 35 min will markedly improve the analyses and information content of in vivo measurements.

**Supplementary Information:**

The online version contains supplementary material available at 10.1186/s12968-021-00725-4.

## Background

Atherosclerosis is a complex, chronic inflammatory disease of large and medium sized arteries, characterized by the formation of intimal lesions (atherosclerotic plaques) [1]. These plaques mainly occur at bifurcations, branching regions and in the inner curvature of the aortic arch, where the wall shear stress (WSS) is low and oscillatory [2–4]. Another main pathophysiological feature of atherosclerosis is the gradual arterial stiffening due to the loss of elastic fibers [5] and an increased collagen deposition during plaque development [6]. This increasing stiffness is one of the earliest markers of functional and structural changes and can be characterized by measuring the pulse wave velocity (PWV), which is defined as the speed of arterial pressure waves travelling along the aorta and large arteries [7]. An altered WSS profile also appears to correlate with local elastic changes in the vasculature [8]. As hemodynamic parameters like the WSS play a key role in local plaque development [8], and as ultrasound-measurements indicate a possible link between higher PWV and lowered WSS [9], the simultaneous measurement of these two parameters is of great interest. In comparison to ultrasound, cardiovascular magnetic resonance (CMR) provides better tissue contrast and higher dimensional flow measurements. Therefore, quantifications of aortic PWV and WSS with flow sensitive 4D-phase contrast (PC) CMR are potential tools for preclinical research and early diagnosis of atherosclerosis. Ultimately, PWV and WSS may serve as CMR-based biomarkers of the vascular state and atherosclerotic lesion burden.

The widely used ApoE^−/−^ mouse represents a good model for atherosclerosis research [10]. By feeding a high fat and cholesterol-rich diet, the development of atherosclerotic plaques can be accelerated to investigate the relationship of hemodynamic parameters like PWV and WSS in atherogenesis in a reasonable time frame.

The simultaneous measurement of both WSS and PWV parameters is challenging. While a high spatial resolution is required to determine the velocity gradients at the vessel wall for WSS quantification [11], high temporal resolution is needed to accurately capture the rapid propagation of the pulse wave [12, 13]. Thus, in most cases two separate measurements are conducted to measure both parameters, which prolongs acquisition time. For combined measurements of both cardiovascular parameters in swine, Wentland et al. developed an undersampled radial 4D-PC cine sequence with retrospective electrocardiogram (ECG) gating. It was shown that a single 4D-PC CMR measurement was sufficient to determine both PWV and WSS in a scan time in the order of 10 min [14]. The main challenges to adopt this method for measurements in murine models, however, are the small vessel dimensions (~ 10 times smaller) and the high heart rates (~ 5 times higher) compared to porcine models [14, 15]. Since the repetition times and relaxation times of CMR measurements in mice is on the same scale as for acquisitions in swine and cannot be shortened significantly, post processing techniques need to be adapted to fulfill the demands of PWV and WSS quantification in rodents. Zhao et al. presented a first approach to measure both parameters with a radial ECG- triggered technique in the abdominal aorta of mice [16], however, this technique relies on measuring flow in 2-dimensional planes. For a full coverage of flow dynamics in curved vessels such as the aortic arch, a full 3D measurement is needed [17]. Retrospective navigation and radial trajectories may be suitable for this task due to the highly flexible reconstruction framework [18]. For example, view-sharing reconstructions can be optimized to either reconstruct images at higher spatial resolution and lower frame rates or vice versa. Therefore, image reconstruction can be adjusted individually to the demands of a specific parameter. Recently we presented a radial 4D-PC cine CMR sequence for self-gated flow and WSS measurements in the murine aorta at high spatial and moderate temporal resolution [15]. The goal of this work is to further investigate the capabilities of the sequence and the retrospective reconstruction framework by modifying the post-processing algorithms to enable arbitrary frame rates and variable spatial resolutions. The new technique is utilized to extract both PWV and WSS from the same measurement and to investigate the influence of temporal and spatial resolution. Since both parameters are assessed in an acquisition time of only 35 min, the duration of in vivo scan protocols for preclinical studies is significantly reduced. This is demonstrated in an in vivo study using healthy wildtype and atherosclerotic ApoE^−/−^ mice.

## Methods

### Animal handling

Female wildtype (WT) C57BL/6 J mice (n = 5) and ApoE knock-out mice (ApoE^−/−^, n = 5) (both 18 weeks old and from Charles River Laboratories, Sulzfeld, Germany) were fed a normal diet (WT) or a western type diet (ApoE^−/−^: ssniff, Soest, Germany) for 14 weeks. Mice were anesthetized by a nose cone, applying 1.5% isoflurane in 2.0 Vol. % oxygen (2 L/min). A pressure-sensitive pneumatic balloon (Graseby Medical Ireland Limited, Dublin, Ireland) was used for cardiac and respiratory monitoring in real-time [15] by a custom-built ECG unit [19]. All animal experiments were approved by local authorities (Regierung von Unterfranken, Würzburg, Germany, reference number: 55.2-2531.01-427/17) to comply with German animal protection law.

### Data acquisition and processing

Measurements were performed with a 17.6 T vertical bore small animal CMR system (Bruker Avance 750 WB, Bruker BioSpin MRI GmbH, Rheinstetten, Germany) with a 1 T/m gradient system (diameter: 40 mm) and a custom-built single-channel transmit-receive electromagnetic (TEM) resonator (inner diameter: 24 mm). Flow was quantified in the aortic arch using a slab-selective non-triggered radial 4D-cine phase contrast sequence, as proposed recently [15]. Spatial encoding was performed using a 3D radial trajectory with an angular density adjusted to the anisotropic field of view (FOV) [20]. In addition, a 3D B_0_ map was acquired for subsequent off-resonance correction [15]. All relevant scan parameters can be found in Table 1. Data processing and reconstruction was performed with Matlab (version 2016b, Mathworks, Inc., Natick, Massachusetts, USA). Self-navigation signals were extracted as proposed in [15, 21, 22].

**Table 1:** Scan and reconstruction parameters for the 4D-flow measurement

Parameter	HS/LT-res. reconstruction	LS/HT-res. reconstruction
Repetition time [ms]	3.0	3.0
Echo time [ms]	1.1	1.1
Flip angle [°]	15	15
Number of readout points	140	140
Number of projections	1.6 ∙ 10^5^	1.6 ∙ 10^5^
Number of velocity Encoding steps	4 (balanced 4-point encoding)	4 (balanced 4-point encoding)
Encoding velocity [cm/s]	125	125
Field of view [mm^3^]	25 × 25 × 4	25 × 25 × 4
Slab thickness [mm]	4	4
Measurement time [min]	32 (+ 3 min for B_0_ map)	32 (+ 3 min for B_0_ map)
Spatial resolution	100 µm	147 µm
N frames per cycle	20	200
Selection window	1/20	1/33

*HS/LT* high spatial/low temporal, *LS/HT* low spatial/high temporal

### WSS analysis

#### Reconstruction

**Fig. 1:**
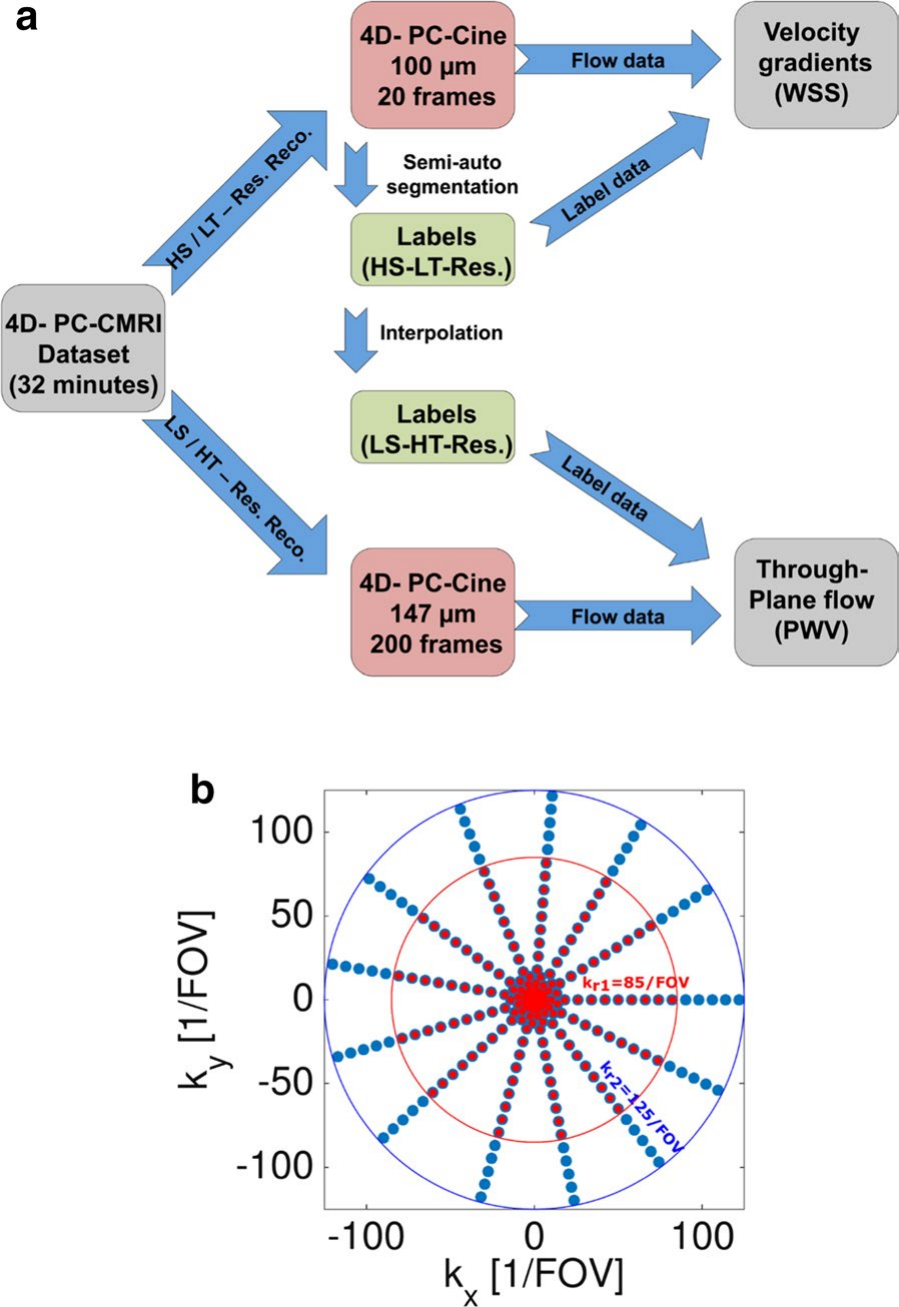
**a** An Illustration of the reconstruction process for the WSS- and PWV evaluation. For WSS calculation, the 4D-PC data was reconstructed at high spatial and low temporal resolution (HS/LT-res. reconstruction, see top panels). The aortic lumen was labeled with semi-automatic segmentation. Using the flow and label data, velocity gradients were calculated. For PWV calculation, the 4D-PC data was reconstructed at low spatial and high temporal resolution (LS/HT-res. reconstruction, see bottom panels). The segmentation data obtained from the HS/LT-res. reconstruction was temporally interpolated. Using the interpolated label and flow data, through plane flow was determined at multiple locations. **b** For the LS/HT-res. reconstruction (PWV), only the inner k-space points with kr < 85/FOV were used (red points, shown in 2D for better illustration). The HS/LT-res. reconstruction uses all available k-space frequencies (red and blue points)

**Fig. 2:**
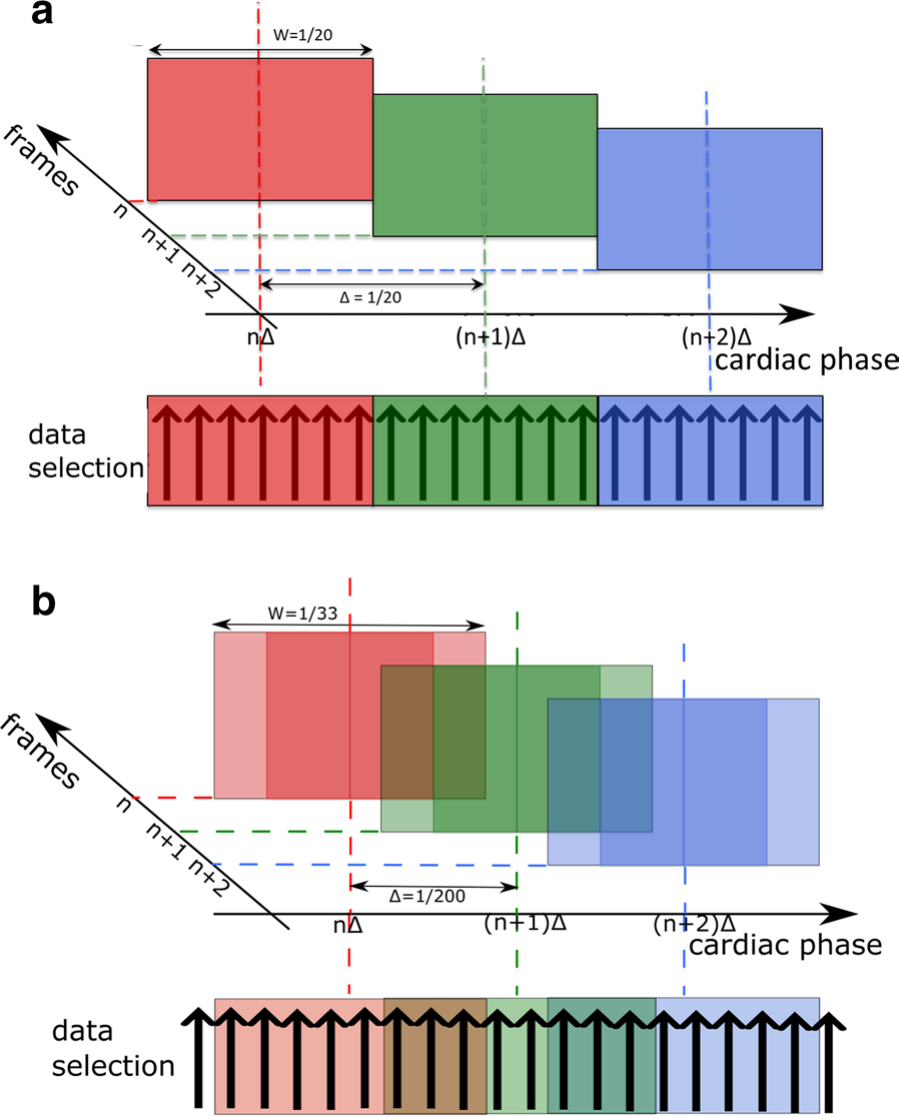
**a** Sliding window selection of cardiac phases for the HS/LT-resolution reconstruction (illustrated for only 3 frames and unscaled for a better clarity). The selection window width of the average heart cycle was W = 1/20 and the distance between each window Δ = 1/20. **b** Sliding window selection of cardiac phases for the LS/HT- resolution reconstruction. Overlapping selection windows with a width of W = 1/33 and a distance of Δ = 1/200 of the average cardiac cycle were used. The lighter shadings at the left and right edges of the selection windows mark the overlapping areas (not at scale)

For the WSS in vivo study, 3D-cines were reconstructed retrospectively at high spatial and lower temporal resolution (HS/LT-res. reconstruction, see Fig. 1a). To achieve maximum spatial resolution, all available radial k-space points were considered for reconstruction (see Fig. 1b, red and blue points). The cardiac cycle was divided into 20 data selection intervals, corresponding to 20 relative phases in the heart cycle (see Fig. 2a), as recently proposed [15]. The selection windows had a width of W = 1/20 of the full heart cycle, which corresponds to an average of 5–7 ms. Four 3D-cines (one flow-compensated and three flow-encoded) with an isotropic spatial resolution of 100 µm, respectively, were reconstructed with a Non-Uniform Fast Fourier Transform (NUFFT) [23, 24].

#### WSS calculation

The lumen of the aortic arch was segmented with a semi-automatic labeling technique [25] and velocity was calculated from the phase differences of the four encoders [15]. A custom python software tool developed for Ensight 10.0.2 (Ansys, Inc., Canonsburg, Pennsylvania, USA) was used to calculate the WSS [15].

### PWV analysis

#### Reconstruction

PWV was determined with the multiple-points transit-time method [13, 16], which requires higher frame rates in comparison to the WSS measurement [14]. Hence, the reconstruction algorithm was adapted to reconstruct the same 4D flow data at lower spatial and higher temporal resolution (LS/HT-res. reconstruction, see Fig. 1a). Lower spatial resolution was chosen in order to minimize undersampling artifacts and noise. Therefore, only the inner 64.5% of the original data points (Fig. 1b, red points) were used for reconstruction. The high effective frame rate (defined by the number of reconstructed frames covering the complete cardiac cycle) was achieved by applying overlapping sliding windows (see Fig. 2b). Each window had a width of W = 1/33, which corresponds to 3–4 ms (approximately 1 × TR). Using this kind of data selection, the relative phases were divided into 200 intersecting intervals (relative distance between each interval: ∆ = 1/200 of the average heart cycle, or 0.5–0.7 ms). The associated projections within each interval were combined for image reconstruction. After rigid motion and off-resonance correction [15], a set of four 3D-cines (native spatial resolution 147 µm isotropic, 200 frames, respectively), was reconstructed and afterwards zero-filled to an isotropic spatial resolution of 74 µm.

#### PWV calculation

**Fig. 3:**
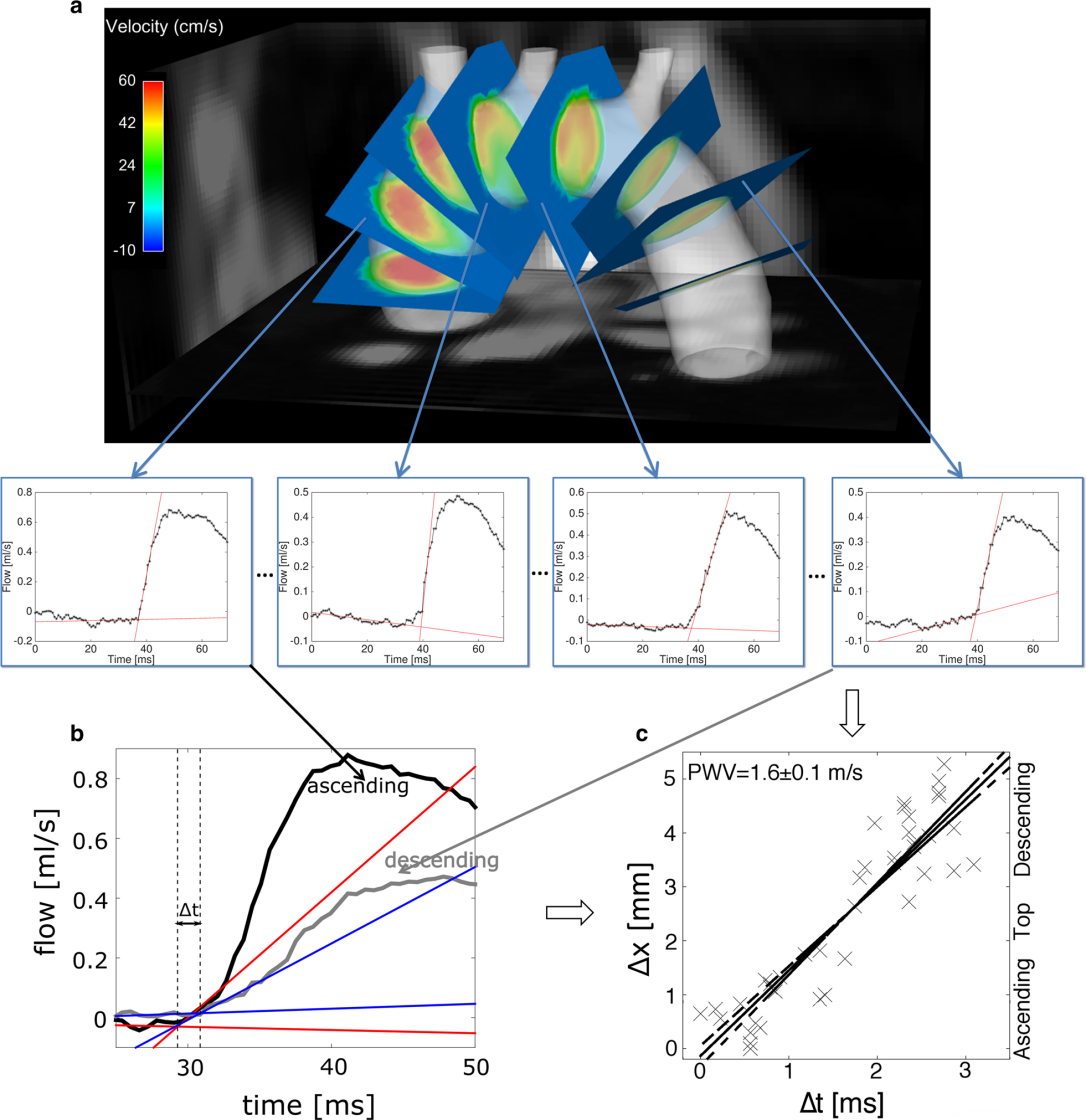
**a** Illustration of the transit time pulse wave velocity (PWV) measurement. Through-plane flow was determined at various positions along the aortic arch (for illustration only 8 planes are shown; in the experiment approx. 50 slices were used). **b** For each location (here shown for one plane in the ascending and descending aorta), the time point of the systolic upstroke was identified as the intersection of a line fitted to the pre-systolic data points and a line fitted to the upstroke of the early systolic pulse. **c** The distances Δx of each plane (relative to the proximal ascending aorta) were plotted against the time points of the systolic upstroke Δt (relative to the time point of the first plane). PWV was calculated from the slope of a line fitted to this plot

The multiple-points transit-time method relies on accurately determining the timing of the upstrokes of through-plane flow curves. For analysis of velocity data, the segmentation was temporally interpolated with linear interpolation and re-gridded to the spatial resolution of the PWV reconstruction. The labeled data was used to set the velocity field outside the aorta to zero. Subsequently, the velocity data was smoothed with a 3-connectivity neighborhood spatial median filter and exported to EnSight. Using python software (Ansys, Inc), a centerline of the lumen segmentation was calculated. Through-plane flow was afterwards determined at approximately 50 equidistant locations (except regions close to the aortic branches) along the aortic arch and perpendicular to the centerline (see Fig. 3a). Subsequently, the flow curves were imported to Matlab (Mathworks, Inc.) for further data processing. For each plane, the time point of the systolic upstroke of the volume flow was identified by measuring the intersection of a line fitted to the pre-systolic data points (baseline) and a line fitted to the upstroke of the early systolic pulse (Fig. 3b). For more precise determination of the time point of the systolic upstroke, a tenfold linear interpolation was applied to the flow data prior to fitting. Subsequently, the distances of each plane Δx (relative to the proximal ascending aorta) were plotted against the time points of the systolic upstroke Δt (Fig. 3c). For PWV calculation, a line was fitted to the plot. PWV was afterwards derived from the slope of this fit:

1 $$PWV= \frac{dx}{dt}$$

### Yield, undersampling and temporal blurring

To assess the quality of self-navigation, the yield (i.e. the number of detected heartbeats N relative to the theoretically expected total number of heartbeats N_th_ occurring during a measurement time of 32 min) was calculated:

2 $$Yield\left[\%\right]=\frac N{N_{th}}\cdot100=\frac{N\cdot{\overline T}_{RR}}{32min}\cdot100$$

Hereby ${\overline{T}}_{RR}$ denotes the average cardiac period, which is determined with the self-navigation signal. Since the cardiac signal is affected by respiratory motion, the yield is usually smaller than 100%.

In order to estimate the degree of undersampling according to the Nyquist criterion, the theoretical number of projections N_Nyq_, necessary to reconstruct an artifact-free image, was calculated. For an isotropic image matrix of the dimensions J × J × J, a field of view of 25 × 25 × 25 mm^3^ and a slab thickness of 4 mm, N_Nyq_ can be approximated as [26]:

3 $$N_{Nyq}\approx\pi\cdot J^2\cdot\frac4{25},$$

Since only a thin slab is excited and the trajectory is adjusted for reconstruction of an anisotropic FOV, the necessary number of projections is reduced by a factor of $\frac{4}{ 25}$. Equation (3) results into N_Nyq_ $\approx$ 1.4 · 10^4^ for the LS/HT-res. reconstruction and N_Nyq_ $\approx$ 3.1 · 10^4^ for the HS/LT-res. reconstruction, respectively. The undersampling factor can be afterwards calculated using:

4 $$us= \frac{{N}_{Nyq}}{{\overline{N}}_{f}} ,$$

where $\overline{N}_{f}$ is the average number of projections available to reconstruct a cine frame.

For the assessment of the temporal accuracy of the reconstructions, the standard deviations *δ*p of the binned relative phases were calculated for each selection interval (Fig. 2a and b). The temporal blurring is defined as $\delta t=\delta\overset-p\cdot{\overset-T}_{RR}$, where $\delta \stackrel{-}{p}$ is the mean standard deviation of relative phases averaged over all selection intervals and ${\stackrel{-}{T}}_{RR}$ the temporally averaged cardiac period.

### Temporal and spatial resolution

To investigate the influence of spatiotemporal resolution, one in vivo measurement was reconstructed at different frame rates (20–200 frames / heart cycle) and spatial resolutions (100 µm, 125 µm, 147 µm) as well as varying selection window widths (1/20–1/60), respectively. PWV and the time-dependent WSS magnitude values were assessed from these generated cines. Each PWV value was determined using the same analysis planes for the through-plane flow assessment as for the LS/HT-res. reconstruction. Prior to the PWV determination, each extracted through-plane flow curve was interpolated to the same effective temporal resolution as the 200 frames reconstruction using spline interpolation.

### Error and statistics

All error and statistical analyses were performed in Matlab (version 2016b,  Mathworks, Inc) and Prism 7 (GraphPad Software, San Diego, California, USA). In order to assess the PWV error, the standard deviation of the linear fit and the coefficient of determination R^2^ were calculated. The Shapiro Wilk Normality Test was used for testing normal distribution. For normally distributed data, an unpaired sample t-test was utilized [27]. When normality test failed, a non-parametric Mann–Whitney-U test was performed. For analysis of the degree of correlation between heart rates with other parameters, the Pearson correlation coefficients were calculated. Differences of p < 0.05 were considered to be statistically significant.

## Results

### Stability of self-navigation

**Fig. 4:**
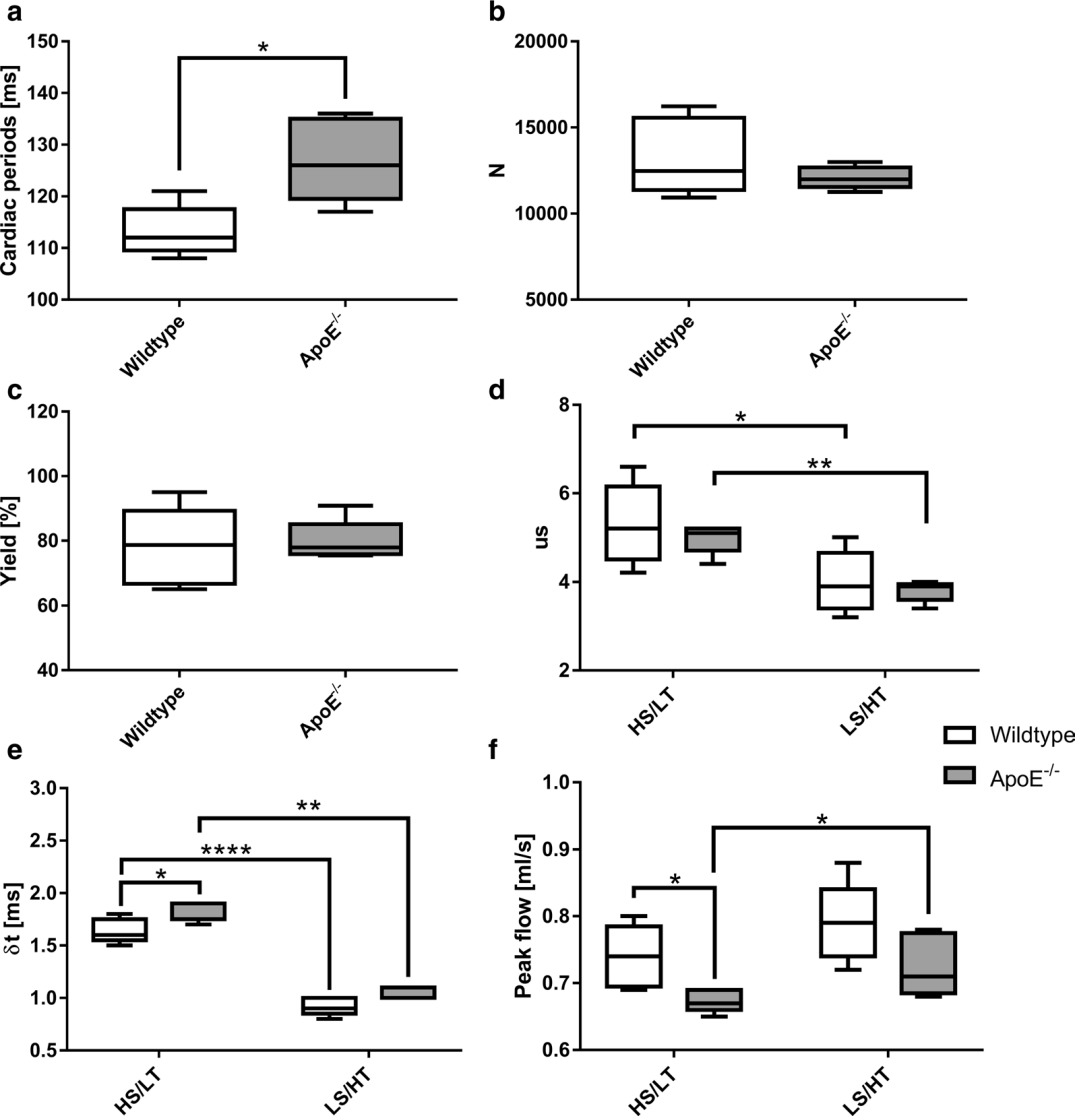
Stability of self-navigation, undersampling and temporal blurring. **a** Cardiac periods T_RR_ for wildtype and ApoE^−/−^ mice. A slight significance was observed between both groups (*, p = 0.01). **b** No significant differences could be observed in the total number N of detected heartbeats. **c** Yield for both groups, showing no significant differences. **d** Undersampling factors for the high spatial/low atemporat (HS/LT) and low spatial/high temporal (LS/HT) reconstructions in both animal groups (Wildtype: (HS/LT vs. LS/HT, *, p = 0.03. ApoE^−/−^: HS/LT vs. LS/HT, **, p < 0.01). **e** Temporal blurring for the HS/LT and LS/HT reconstructions in both animal groups (HS/LT: Wildtype vs. ApoE-/- *, p < 0.04. Wildtype: HS/LT vs. LS/HT, ****, p < 0.0001. ApoE^−/−^: HS/LT vs. LS/HT, **, p < 0.01). **f** Peak flow values for the HS/LT and LS/HT reconstructions in both animal groups (HS/LT: Wildtype vs. ApoE^−/−^, *, p = 0.02. ApoE^−/−^: HS/LT vs. LS/HT, *, p = 0.05)

**Fig. 5:**
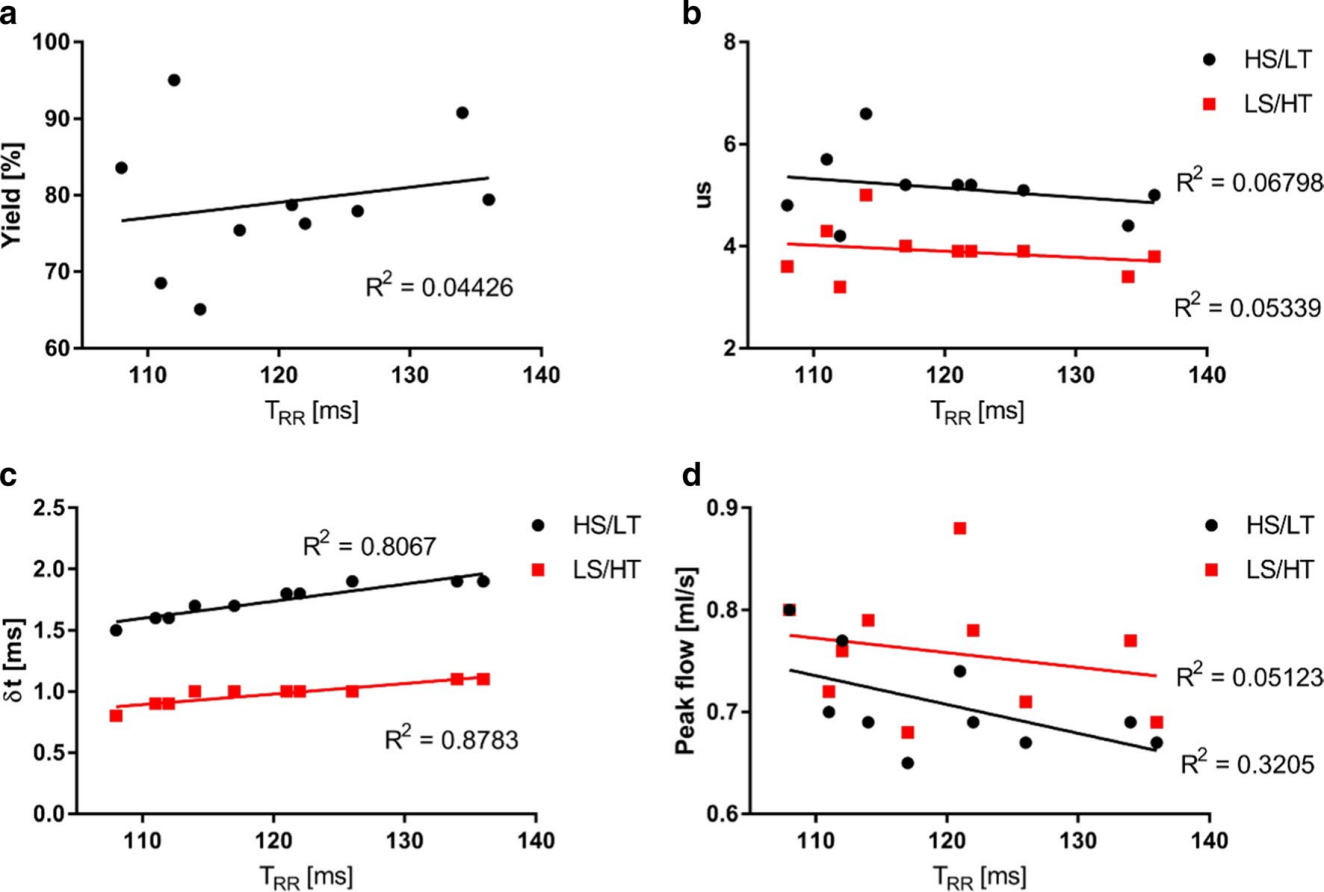
Correlation plots of cardiac periods with different parameters. **a** No dependency of the yield on the heart rate was observed (p = 0.56). **b** No correlation was observed between undersampling and heart rates (HS/LT: p = 0.47, LS/HT: p = 0.52). **c** A correlation was observed between the temporal blurring and the heart rate (HS/LT: p < 0.0001, LS/HT: p < 0.001) due to the relation of the width of the selection window with the cardiac periods. **d** In both reconstructions, no significant correlation between the cardiac periods and peak flow values was observed (HS/LT: p = 0.09, LS/HT: p = 0.53)

Self-navigation signals could be obtained from both the ApoE^−/−^ and WT group. Figure 4a shows the results for the mean cardiac period $\overline{T}_{RR}$ , averaged over all heartbeats detected during the 32 min of measurement. The standard deviations for the ApoE^−/−^ and WT group are ± 4 ms and ± 8 ms, respectively. Cardiac periods were mildly elevated in the atherosclerotic mouse model (p = 0.01, see Fig. 4a). The total number of heartbeats N detected by the algorithm and the yield (see Eq. (2)) were determined (Fig. 4b, c). The yield (Fig. 4c) was independent of the heart rate (see Fig. 5a). In average, approximately 20% of the data was omitted due to respiratory gating in both subsequent reconstructions.

### Undersampling and temporal blurring

Figure 4d displays the undersampling factors (see Eq. (4)) for both types of reconstructions. In the HS/LT-res. reconstruction, the mean undersampling factor was 5.3 ± 0.9 for WT mice and 5.0 ± 0.4 for ApoE^−/−^ mice, respectively. In the LS/HT-res. reconstruction, undersampling was 4.0 ± 0.7 for WT mice and 3.8 ± 0.2 for ApoE^−/−^ mice. Less undersampling was achieved in the reconstruction with high temporal resolution due to the 32% lower spatial resolution. No correlation was observed between undersampling and heart rates (HS/LT: p = 0.47, LS/HT: p = 0.52, see Fig. 5b).

Figure 4e and Fig. 5c show the mean temporal blurring (see above) of the reconstructed 4D-PC-cine frames. In the HS/LT-res. reconstruction, the mean temporal width was 1.5–1.8 ms. In the LS/HT-res. reconstruction, the temporal blurring was 1.8–2 times smaller. Since the width of the selection windows is related to the cardiac period, a correlation was observed between the temporal blurring and the heart rate (HS/LT: p < 0.001, LS/HT: p < 0.001, see Fig. 5c). Larger temporal blurring was detected in the HS/LT reconstruction of ApoE^−/−^ mice (p = 0.04) due to the lower heart rate, however, no significant differences were found in the LS/HT reconstruction (p = 0.08, see Fig. 4e).

### Flow values

**Fig. 6:**
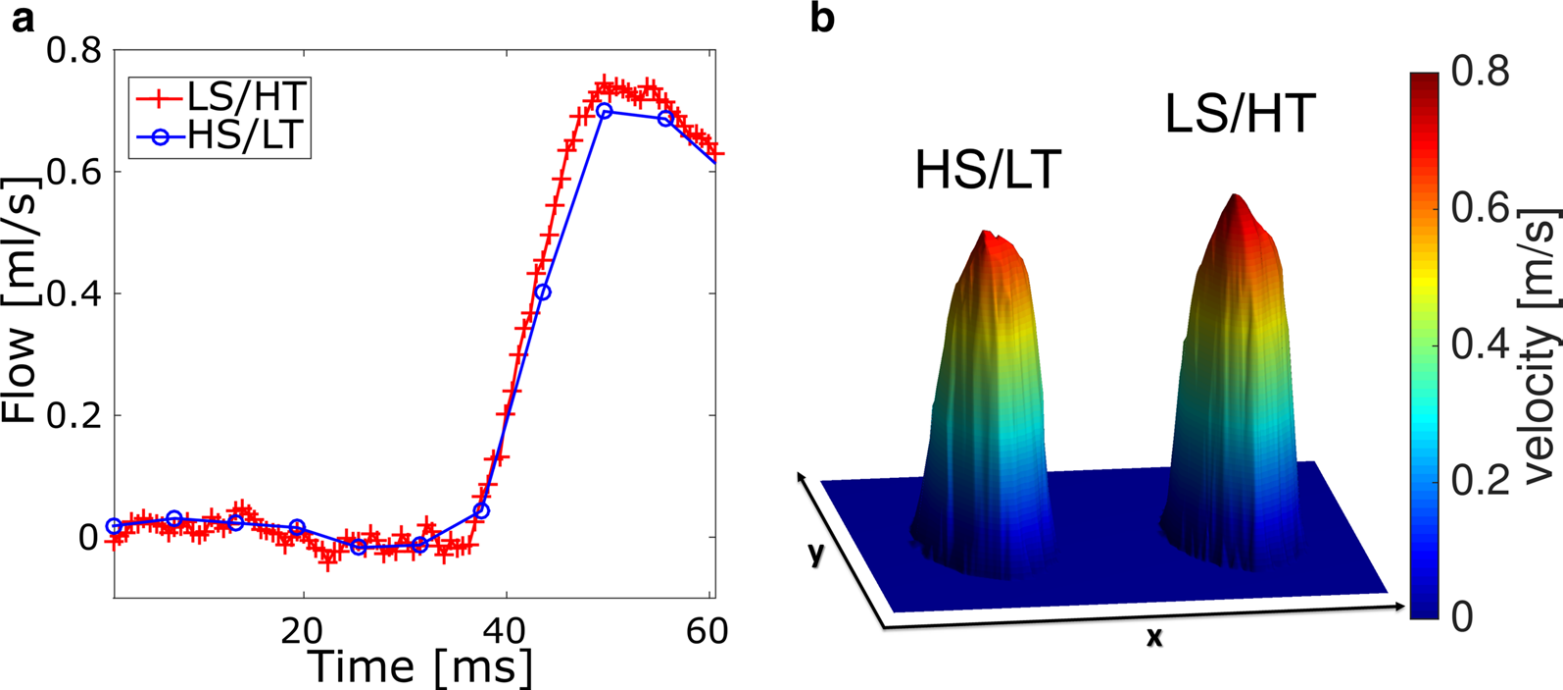
**a** Through-plane flow in the ascending aorta of an ApoE^−/−^ mouse, determined with the HS/LT-resolution (blue) and LS/HT-resolution (red) reconstruction. **b** Peak flow profile of the same plane (HS/LT and LS/HT reconstruction)

Figure 6a shows two reconstructions of through-plane flow in the ascending aorta of a representative ApoE^−/−^ mouse, obtained from an LS/HT-res. and an HS/LT-res. reconstruction, respectively. Figure 6b displays the corresponding peak flow profiles. In the reconstruction with higher temporal resolution, the upstroke of the flow pulse is clearly recognizable and larger peak flow values are noticeable. Figure 4f shows the corresponding peak flow values for all animals. At higher temporal resolution, an increase in peak flow values is noticeable. In both reconstructions, no significant correlation between cardiac periods and peak flow values was observed (HS/LT: p = 0.09, LS/HT: p = 0.53, see Fig. 5d).

### Influence of temporal and spatial resolution

#### Pulse wave velocity

Table 2 displays the PWV values obtained from different reconstructions of the same in vivo experiment (see also Additional file 1: Figs S1–S3 in the supplement for the individual fits). In the first step, the frame rate of the LS/HT-res. reconstruction was artificially reduced to 100 and 50 frames by taking only every second and fourth frame of the reconstruction, respectively. The results suggest an increased PWV error and a lower R^2^ value when only 50 frames are used (see Table 2, I). The 100 frames analysis already results in a much better accuracy of the fit and thus a lower PWV error. The PWV, however, is slightly underestimated in comparison to the full 200 frames dataset. In the next step, 4D flow cines were reconstructed using varying frame rates and window widths with a spatial resolution of 100 µm (see Table 2, II). The PWV value obtained from the cine with 20 frames and a 1/20 window width features a comparably large error relative to the LS/HT-res. reconstruction and a low coefficient of determination (R^2^ = 0.59).

**Table 2:** PWV estimation at different spatial resolutions, frame rates and selection window widths

Resolution [µm]	Frames	Width	PWV [m/s]		Error [m/s]	R^2^	US
**I. Influence of frame rates **							
147	50 OV	1/33	1.50		0.13	0.77	3.6
147	100 OV	1/33	2.00		0.06	0.97	3.6
147	**200 OV (LS/HT)**	1/33	2.44		0.10	0.93	3.6
**II. Influence of window widths**							
100	**20 (HS/LT)**	1/20		2.40	0.28	0.59	4.8
100	30	1/30		2.05	0.15	0.77	7.2
100	40	1/40		2.30	0.11	0.89	9.6
100	50	1/50		1.85	0.49	0.21	12.0
100	60*	1/60		–	–	–	14.4
100	60 OV	1/20		1.66	0.16	0.68	4.8
**III. Influence of spatial resolution**							
100	200 OV	1/33	2.52		0.18	0.78	7.8
125	200 OV	1/33	2.20		0.08	0.93	5.0

*OV* reconstructions with overlapping frames. R^2^: Coefficient of determination. us: undersampling factor. In bold: measurements used for the later PWV (LS/HT) and WSS (HS/LT) analysis. *No data evaluation possible. For the individual fits, see Additional file 1: Figs. S1–S3 in the supplement

A better accuracy was observed when larger frame rates and smaller selection windows were used. However, in the cine reconstructions with 50 frames or higher, severe undersampling artifacts occurred that precluded an accurate PWV measurement. The determination of a PWV value was not possible for a window width of 1/60 and an undersampling factor of more than 14. To further increase the frame rates, a cine with 60 overlapping frames and a window width of 1/20 was generated. The results indicated a lower PWV error in comparison to the 20 frames reconstruction but lower accuracy relative to the reconstructions with 100 frames or higher. Finally, 4D flow cines were generated at different spatial resolutions and 200 overlapping frames (window width: 1/33, see Table 2, III). While the results of the 125 µm reconstruction were similar to the 147 µm cine, a larger PWV error was observed in the 100 µm reconstruction due to more prominent undersampling artifacts.

#### Wall shear stress

**Fig. 7:**
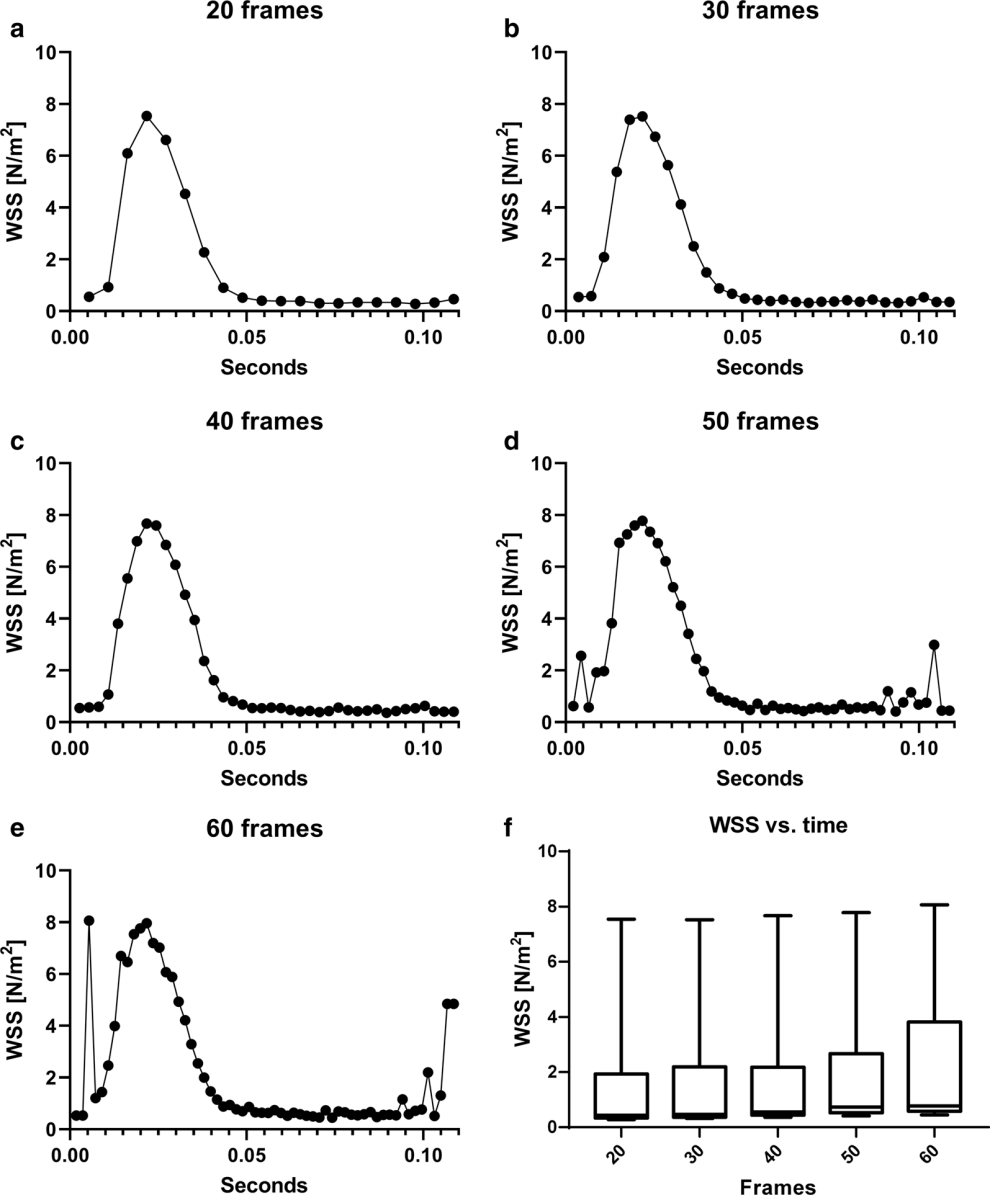
Time-dependent median WSS values for different temporal resolutions. **a** WSS values with 20 frames. **b** WSS values with 30 frames. **c** WSS values with 40 frames. **d** WSS values with 50 and 60 (**e**) frames. In **d** and **e**, severe undersampling factors are noticeable. **f** Boxplots of the WSS distribution over the cardiac cycle for all reconstructions. A slight increase of peak WSS values compared to the 20 frames reconstruction when using higher frame rates and smaller selection windows is observable

**Fig. 8:**
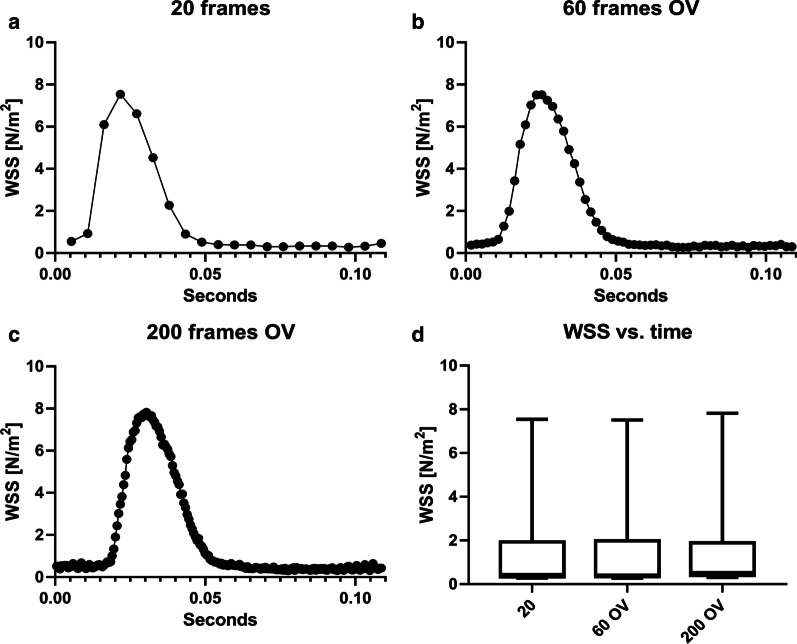
Time-dependent median WSS values for different temporal resolutions with overlapping selection windows. **a** WSS values with 20 frames. **b** WSS values with 60 overlapping (OV) frames. **c** WSS values with 200 overlapping (OV) frames. **d** Boxplots of the WSS distribution over the cardiac cycle for all reconstructions. The reconstruction with the highest frame rate (200 frames, W = 1/33) features a slightly larger peak WSS

The 100 µm 4D cine reconstructions (see Table 2, II) were used to investigate the influence of selection window widths and frame rates on WSS values. Figure 7a–e displays the time-dependent median WSS for different temporal resolutions (20–60 frames). In Fig. 7f, boxplots of the WSS distribution over the cardiac cycle are shown. The results indicate a slight increase of the peak WSS compared to the 20 frames reconstruction when higher frame rates and smaller selection windows are used. However, for window widths of 1/50 and smaller, severe undersampling artifacts were observed. Figure 8 displays the results for reconstructions with overlapping selection windows for 60 overlapping (OV) frames (Fig. 8b) and 200 overlapping frames (Fig. 8c). For comparison, values from the HS/LT-res. reconstruction are shown (Fig. 8a). The reconstruction with the highest frame rate (200 frames, W = 1/33) also features a slightly larger peak WSS (see Fig. 8d).

### In vivo study: WSS values

**Fig. 9:**
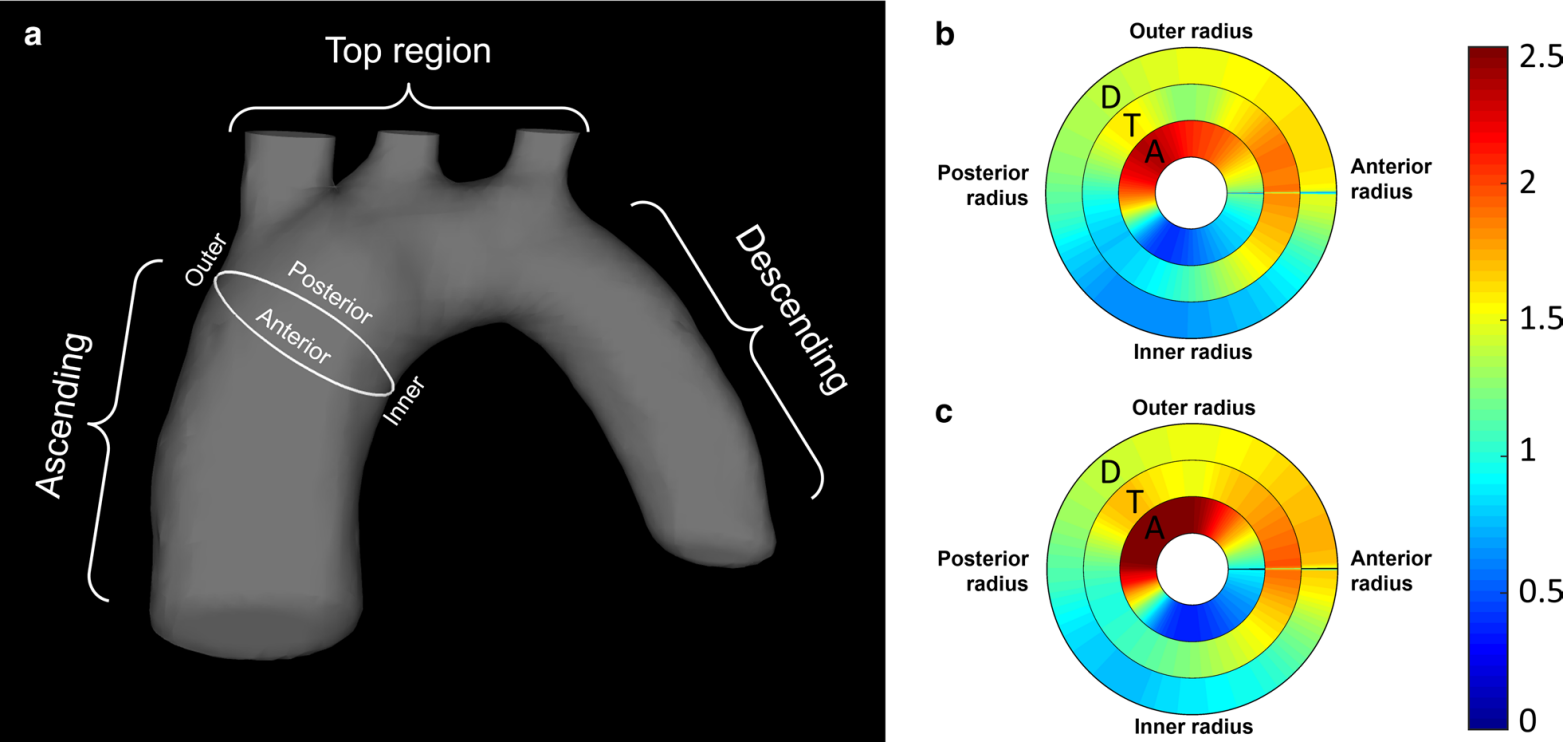
**a** Scheme of the aortic arch with the chosen regions: ascending (A), top region (T) and descending (D). White ring: Scheme of the inner, outer, anterior and posterior radius of the aorta. **b **+ **c** Bulls-Eye plots for the temporal averaged WSS values (magnitude) for **b**: Wildtype and **c**: ApoE^−/−^ mice. Inner ring: Ascending aorta (A). Middle ring: top region (T). Outer ring: descending aorta (D). Scale: N/m^2^

Temporally averaged WSS magnitude values were calculated in the aortic arch of all wildtype and ApoE^−/−^ mice using the HS/LT-res. reconstruction (20 frames). Figure 9 shows bulls-eye plots for the distribution of WSS magnitudes in three regions of interest (inner ring: ascending aorta (A), middle ring: top region (T), outer ring: descending aorta (D), see scheme in Fig. 9a). Mean WSS values averaged over the whole aortic arch were (1.17 ± 0.07) N/m^2^ (WT mice, see Fig. 9b and (1.27 ± 0.10) N/m^2^ (ApoE^−/−^ mice, see Fig. 9c). In both groups, a highly asymmetric distribution of WSS values in the ascending aorta is noticeable. Results also indicate a larger asymmetry of WSS distribution in the ApoE^−/−^ group.

### In vivo study: PWV values

**Fig. 10:**
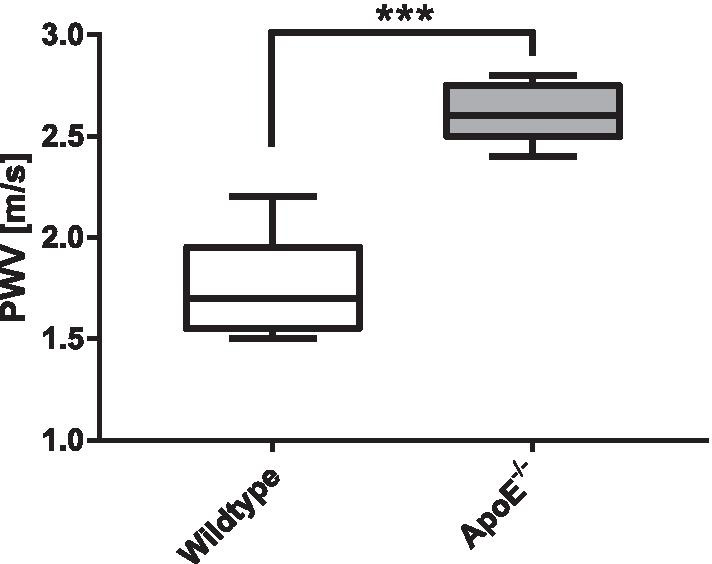
Pulse wave velocities for wildtype and ApoE^−/−^ mice. Data are presented as mean ± SD. Statistically significant differences are observable in ApoE^−/−^ mice compared to the wild type  group (***, p < 0.001)

Aortic PWV was assessed in all 10 animals using the multiple-point transit time method and the LS/HT-res. reconstruction (200 frames). The PWV values are displayed in Fig. 10. In the atherosclerotic ApoE^−/−^ mouse model, significantly higher PWV values were observed compared to the WT control group ((2.6 ± 0.2) m/s versus (1.7 ± 0.2) m/s, p < 0.001).

## Discussion

### Self-gating: stability and undersampling

Analysis of the self-navigation signals showed no significant differences in the heart rate variations as well as in the yield, which suggests no variations in stability between healthy WT and pathological subjects. In both groups, approximately 80% of the radial projections could be used for reconstruction. In the ApoE^−/−^ group, the cardiac periods were slightly longer in comparison to the WT control group. The cause for these differences is unclear and will require further clarification. One possible reason might be that both animal groups were measured on two separate measurement days. For PWV as well as WSS quantification, no external cardiac or respiratory navigation signal was necessary, which strongly facilitates animal handling and reduces the influence of external disturbances, e.g. caused by interferences with the gradient system or a slipping of the sensor probes. Due to the large flip angle, short repetition times and a thin excitation slab, strong signal enhancement of inflowing blood and suppression of static tissue were achieved at the same time. Since the blood signal is orders of magnitudes larger than the signal of the surrounding tissue, undersampling artifacts such as streaking only slightly affect the quality of the reconstructed velocity maps [16]. This allows high acceleration factors with radial PC-MRI [28]. In the reconstruction used for PWV quantification, undersampling factors were between 3.8 and 4.0. In the WSS reconstruction, undersampling factors were larger due to the higher spatial resolution (5.0–5.3). Results were similar to findings reported for WSS measurements in 12-week-old WT mice [15].

### Flow values and segmentation

Peak flow values measured in this study ranged between 0.65 and 0.80 ml/s (HS/LT-res. reconstruction) and between 0.71 and 0.88 ml/s (LS/HT-res. reconstruction), respectively (see Fig. 4f). The results are in good accordance with the values reported previously for 12-week-old WT mice (0.8–0.9 ml/s, [15]). In both animal groups, a 7% increase of peak flow was observed in the LS/HT-res. reconstruction, which can be attributed to the higher frame rate and the smaller selection windows. Furthermore, only the high frame rate reconstruction was capable of accurately detecting the upstroke of the systolic flow pulse. On the other side, only the high spatial resolution reconstruction enables accurate measurements of the velocity gradients and therefore WSS [11]. Thus, a combined analysis of flow at two different time scales and spatial resolutions provide better insight in the complex fluid dynamics of the aortic arch, which will be beneficial for future simulation-based computational fluid dynamics (CFD) studies, especially in the context of atherogenesis [29–31].

For the LS/HT-resolution through-plane flow measurements, interpolated label data were used in order to reduce the time investment of the lumen segmentation. This was justified by the assumption that velocity values of voxels close to the aortic boundaries contribute only minimally to the flow integral [13]. This hypothesis should also be valid in vessels with large curvatures, since in case of laminar flow, flow profiles can still be modeled as asymmetric paraboloids [11]. In future studies, advanced segmentation techniques (e.g. based on machine learning) as a replacement of interpolation for more accurate vessel labeling (especially to further optimize WSS quantification) will be investigated.

### WSS values

Mean WSS values averaged over the aortic arch were (1.17 ± 0.07) N/m^2^ in WT mice and (1.27 ± 0.10) N/m^2^ in ApoE^−/−^ mice. The results are in good accordance with previous studies in WT and ApoE^−/−^ mice [15–17]. A strong asymmetric distribution of WSS values was observed in the ascending aorta. The results suggest an increase of WSS near the outer radius as well as a decrease near the inner radius of the ascending aorta in the atherosclerotic mouse model, indicating major hemodynamic and structural changes in this region, as expected [1]. Future studies could focus on detailed analyses of these interrelations.

### PWV values

Aortic PWV values of previous studies in 18 week-old mice obtained with CMR were 1.9–2.2 m/s for WT mice and 2.6−2.7 ml/s for ApoE^−/−^ mice [5, 13], which is in range with the results found in this study ((1.7 ± 0.2) m/s and (2.6 ± 0.2) m/s, respectively). In older ApoE^−/−^ mice (8–9 months), larger PWV values of 3.00−5.84 m/s were found [16, 25, 32].

### Temporal and spatial resolution

The retrospective flow measurements presented in this work can be interpreted as a statistical process, since radial projections belonging to hundreds of heartbeats are combined to one dataset in order to reconstruct an image. The CMR signal (and therefore the self-navigation signal) is acquired with a repetition time of TR = 3 ms, thus the maximal achievable native temporal resolution is limited to this value. For the cine reconstructions, selection windows are used for the retrospective binning of projections. These windows were overlapped in order to realize a higher effective frame rate. Due to the finite width of the selection windows, each frame of the LS/HT-res. reconstruction had a temporal blurring of ± 0.8−1.1 ms (see Fig. 4e), which is approximately 1.5−1.6 times the distance between two successive frames (approximately 0.6 ms). The selection windows act as a moving average filter that smoothens the flow curve [33]. The cut-off frequencies of these temporal low pass filters range between 220 and 650 Hz, depending on the used selection window size (see Additional file 1: Figure S4). In case of the LS/HT-res. reconstruction (W = 1/33), higher dynamic portions of the flow curve with frequencies above 350 Hz are suppressed. This might result in inaccuracies in the determination of the systolic upstroke. However, a Fourier analysis of triggered Cartesian through-plane flow measurements conducted at a native temporal resolution of 1 ms [21] revealed only small (< − 18 dB, see Additional file 1: Figure S5) contributions of frequency components above 300 Hz to the flow pulse. Therefore, applying a smoothing filter with a width of 1/33 only slightly affects the flow curve (see Additional file 1: Figure S6). Furthermore, since the temporal width of the selection windows is related to the average length of the cardiac period, temporal blurring depends on the heart rate. In this study, no significant correlation between the heart rate and peak flow values was observed. Future studies in animals with much slower heart rates (T_RR_ > 150 ms), however, should address this further.

Investigations of the fitting errors and R^2^ values (see Table 2) indicated a reliable detection of PWV when window widths in the range of 1/30 to 1/40 (cut-off frequencies: 300–400 Hz, see Figure S4) are used. Reconstructions with larger windows result in larger inaccuracies of the PWV measurement due to temporal blurring, even when higher frame rates with overlapping selection windows are utilized. On the other hand, reconstructions with window sizes of 1/50 and smaller exhibit large undersampling artifacts, which impede an accurate assessment of the PWV, thus limiting the minimum achievable temporal blurring. Regarding the frame rate, robust PWV detection was achieved for 40 frames or higher, however, the use of at least 100 frames is recommended in order to guarantee a large range of detectable PWV values. Especially in case of older ApoE^−/−^ mice (8–9 months), PWV can be significantly increased to values of 4 m/s and higher [16, 25]. Hence, a very accurate sampling of the flow curves is required.

Regarding spatial resolution, a reliable PWV value was also found for the reconstruction with an isotropic resolution of 125 µm and an undersampling factor of 5. A further reduction to 100 µm and eightfold undersampling, however, lead to larger inaccuracies due to the more prominent undersampling artifacts. These might be reduced using k-t-acceleration techniques for reconstructions [25]. Since the best fitting results were obtained using window widths between 1/30 and 1/40, frame rates of at least 100 frames and slightly lower spatial resolution, cine reconstructions with 200 frames, window sizes of 1/33 and 147 µm spatial resolution were used for all the PWV evaluations in this in vivo study.

In case of the wall shear stress measurement, an increase of peak WSS was detected when smaller window sizes and larger frame rates are utilized. Thus, the use of higher temporal resolution might be beneficial in order to gain a better understanding of the complex dynamics of wall shear stress. However, as an increase in frame rates leads into a much larger segmentation effort, more advanced segmentation techniques (e.g. using machine learning) need to be implemented first. Therefore we used a frame rate of 20 frames/cycle for this in vivo WSS analysis.

### Limitations

A possible error source of the transit-time method are local disturbances of the flow curves, e.g. caused by recirculating flow or signal cancellations due to off-resonances or accelerated flow. These are especially present in the ascending aorta, near the aortic branches and in the thoracic aorta close to the lung and can impede accurate detection of the systolic upstrokes. To address this issue, through-plane flow was measured in at least 50 locations along the aortic arch in order to minimize the influence of individual disturbed measurements. In general, transit-time techniques for PWV quantification are overall more robust than local PWV measurements since they rely more on the measurement of through-plane flow but less on accurate vessel segmentation [12, 13]. In spite of the small bias and the slightly varying temporal blurring (see section above), the presented method is capable of detecting quantitative differences in PWV between two groups with comparable accuracy as in previous studies [5, 13].

One further limitation of this study is the absence of a reference measurement to validate and further optimize the PWV and WSS measurements. Due to the time constraints in in vivo measurements, no additional triggered 3D flow measurement could be performed. In a previous study, however, our group has already successfully demonstrated the feasibility of the retrospective reconstruction framework for self-navigated local 2D PWV measurements [21]. The results were in very good agreement with triggered Cartesian and radial PWV measurements [12]. Regarding the WSS quantification, validation experiments have already been performed in a flow phantom [15].

For more detailed investigations of the measurement errors, the use of CFD will be investigated in the future. Hereby, the flexible reconstruction enabling variable frame rates and spatial resolutions will be beneficial to further optimize the reconstruction parameters.

### Advantages

Measurements of PWV and WSS are challenging due to the different demands on spatial or temporal resolution. Up to now, usually two separate flow measurements are conducted in order to quantify both parameters in mice [17, 25]. Most PWV measurements rely on cardiac and respiratory gating with external trigger signals, which are prone to external disturbances and add several minutes to the already long acquisition time [13]. For local PWV measurements, k-t acceleration techniques as well as retrospective triggering using self-navigation are available [21, 25], however, local PWV cannot be estimated in the aortic arch due to the strong curvature and reflections near the aortic branches [34]. The highly flexible reconstruction framework presented in this study provides a possible solution to this problem, since both parameters can be derived from one single measurement. Furthermore, 3D PWV measurements allow the assessment of through-plane flow at an arbitrary number of analysis planes, in contrast to 2D-flow measurements, where the number of planes is restricted by the measurement time. This reduces the susceptibility to heart rate instabilities, B_0_ inhomogeneities and flow artifacts [14]. Depending on the demands of the parameter of interest, the reconstructions can be optimized for either high temporal or high spatial resolution. The proposed data processing algorithms are easy to implement and do not require extensive computational efforts. Thus, the assessment of both 3D WSS and PWV in the aortic arch is possible in short measurement and computation times.

The in vivo study presented in this work was performed in a 17.6 T small animal CMR system. However, an adaptation of the radial 4D-PC CMR sequence to 7 T scanners with cryogenic receiver coils [35, 36] should be straightforward. Since the radial trajectory used in this method is susceptible to B_0_ inhomogeneities, lower field strengths might be beneficial. Furthermore, the flexible reconstruction framework is not limited to small animal systems and has already been applied to measurements in humans [18, 37]. Ultimately, the flexible reconstruction might be also beneficial for the development of more realistic CFD models in humans and more efficient clinical routines, for example by addressing arrhythmias in greater detail and using dynamic spatiotemporal resolutions.

## Conclusion

In this work, a new post processing method was presented to extract PWV and WSS from a single flow measurement. The proposed technique provides the assessment of both parameters in an acquisition time of only 35 min, and was successfully applied to both healthy WT and atherosclerotic ApoE^−/−^ mouse models. The results indicate no differences in robustness between healthy WT and in pathological mice.

With this new technique, high spatial and temporal resolution information can concurrently be obtained in short measurement and computation times to gain simultaneously insight into 3D WSS and PWV parameters in the aortic arch. This technique may enable new insights into the hemodynamic determinants of atherosclerosis. In particular, it will be interesting to apply WSS and PWV measurements in order to characterize the hemodynamics during plaque development and progression. Ultimately, these parameters may also contribute to monitoring of plaque stability and the identification of vulnerable plaques in the future, questions of high clinical relevance.

## Supplementary Information

**Additional file 1: Figure S1–S3.** Individual t-versus-x fits for the determination of PWV in order to investigate the influence of the window size (Fig. S1), the frame rates (Fig. S2) and spatial resolutions (Fig. S3). **Figure S4.** Magnitude responses of the temporal filtering due to the retrospective binning of projections. The finite width of the selection window acts as a moving average filter that smoothens the flow curve and suppresses higher dynamic portions. The cut-off frequencies of these low pass filters depend on the window size and are in the range between 220 Hz (W = 1/20) and 650 Hz (W = 1/60). **Figure S5.** Frequency analysis of a high-resolution (1 ms) flow measurement. The flow curve (left) was determined using a triggered Cartesian flow-encoding sequence. A Fourier analysis of the frequency spectrum (right) revealed only small contributions of frequency components > 300 Hz. In dashed lines: Cut-off frequency for the 1/33 selection window (approximately 350 Hz). The CMR measurement used for these plots was originally published in 2013 [21]. **Figure S6.** Temporal filtering with a 1/33 window. The smoothing only slightly affects the flow curve. The CMR measurement used for these plots was originally published in 2013 [21].

## Data Availability

Please contact author for data requests.

## Abbreviations

ApoE: Apolipoprotein E
CFD: Computational fluid dynamics
CMR: Cardiovascular magnetic resonance
ECG: Electrocardiogram
FOV: Field of view
HS/LT-res: High spatial/low temporal resolution
LS/HT-res: Low spatial/high temporal resolution
NUFFT: Nonuniform fast Fourier transform
OV: Overlapping
PC: Phase contrast
PWV: Pulse wave velocity
WSS: Wall shear stress
WT: Wild type
