# Supplementary material for: Simultaneous measurements of 3D wall shear stress and pulse wave velocity in the murine aortic arch
Source: J Cardiovasc Magn Reson. 2021 Mar 18;23:34. doi: 10.1186/s12968-021-00725-4 (PMC7972216; doi:10.1186/s12968-021-00725-4)

FIG S1: Influence of window widths

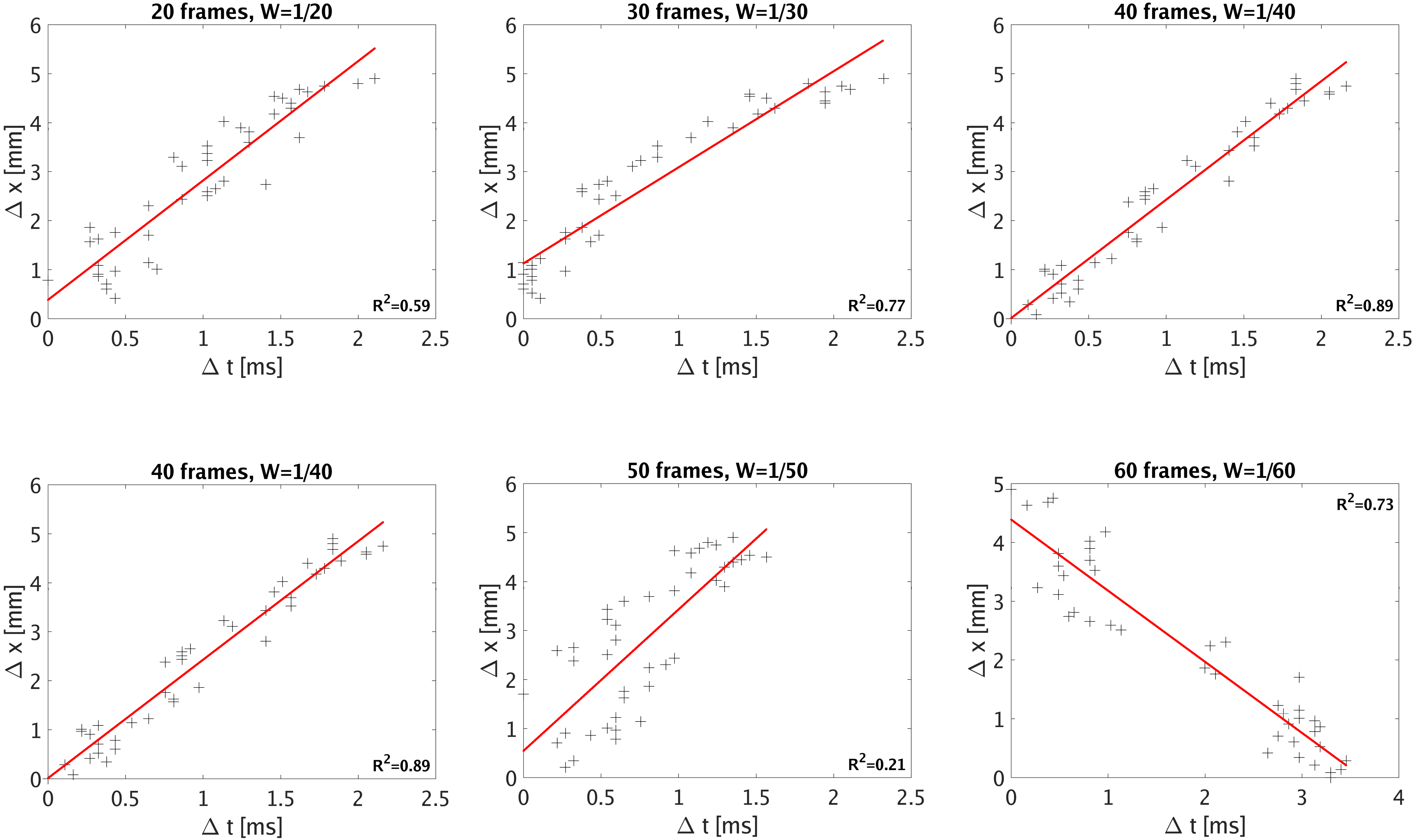

FIG S2: Influence of frame rates

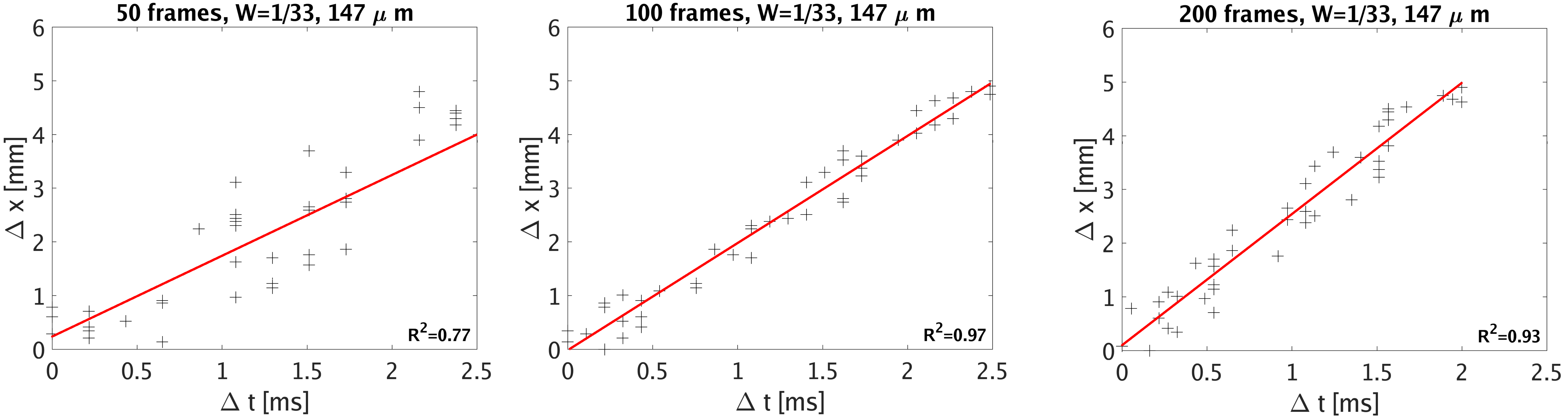

FIG S3: Influence of spatial resolutions

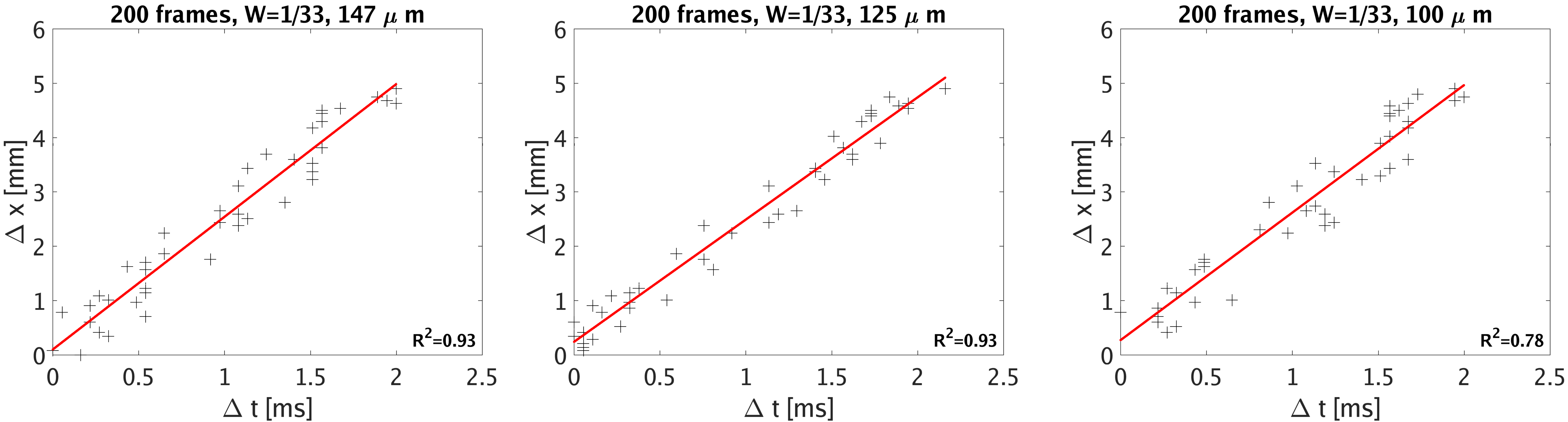

FIG S4: Magnitude Responses of the temporal filtering

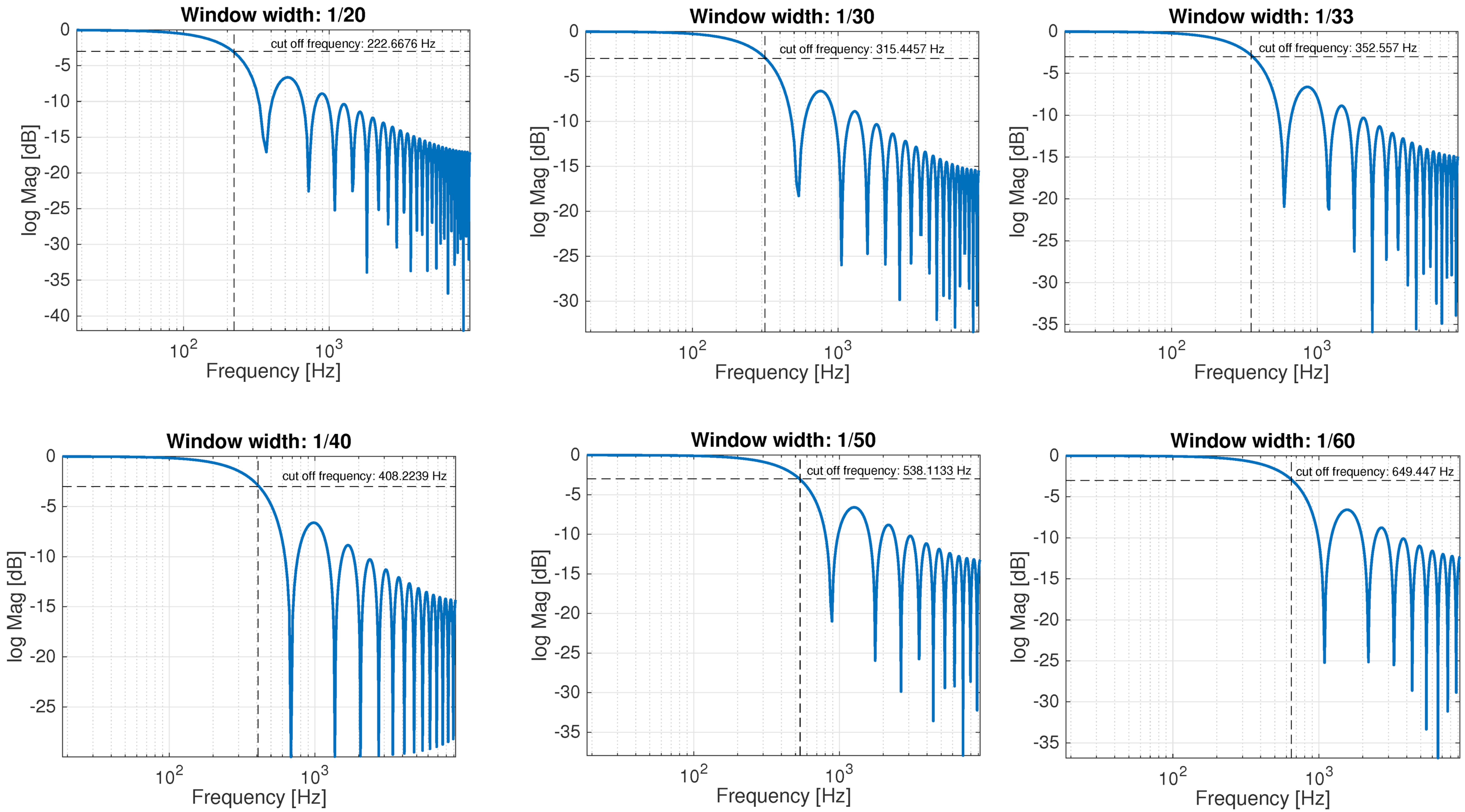

FIG S5: Frequency analysis of a high-resolution (1 ms) flow measurement

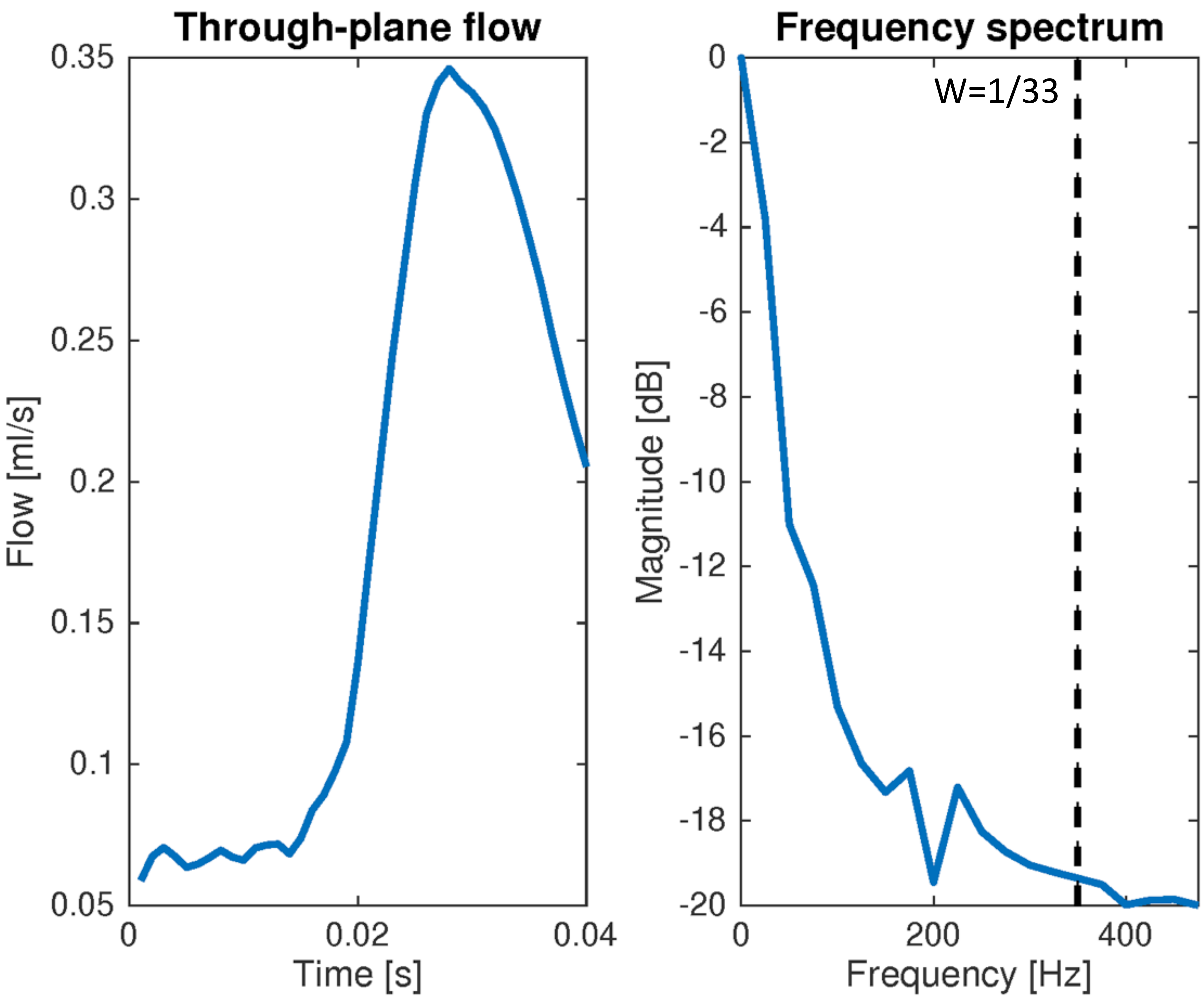

FIG S6: Temporal filtering with a 1/33 window

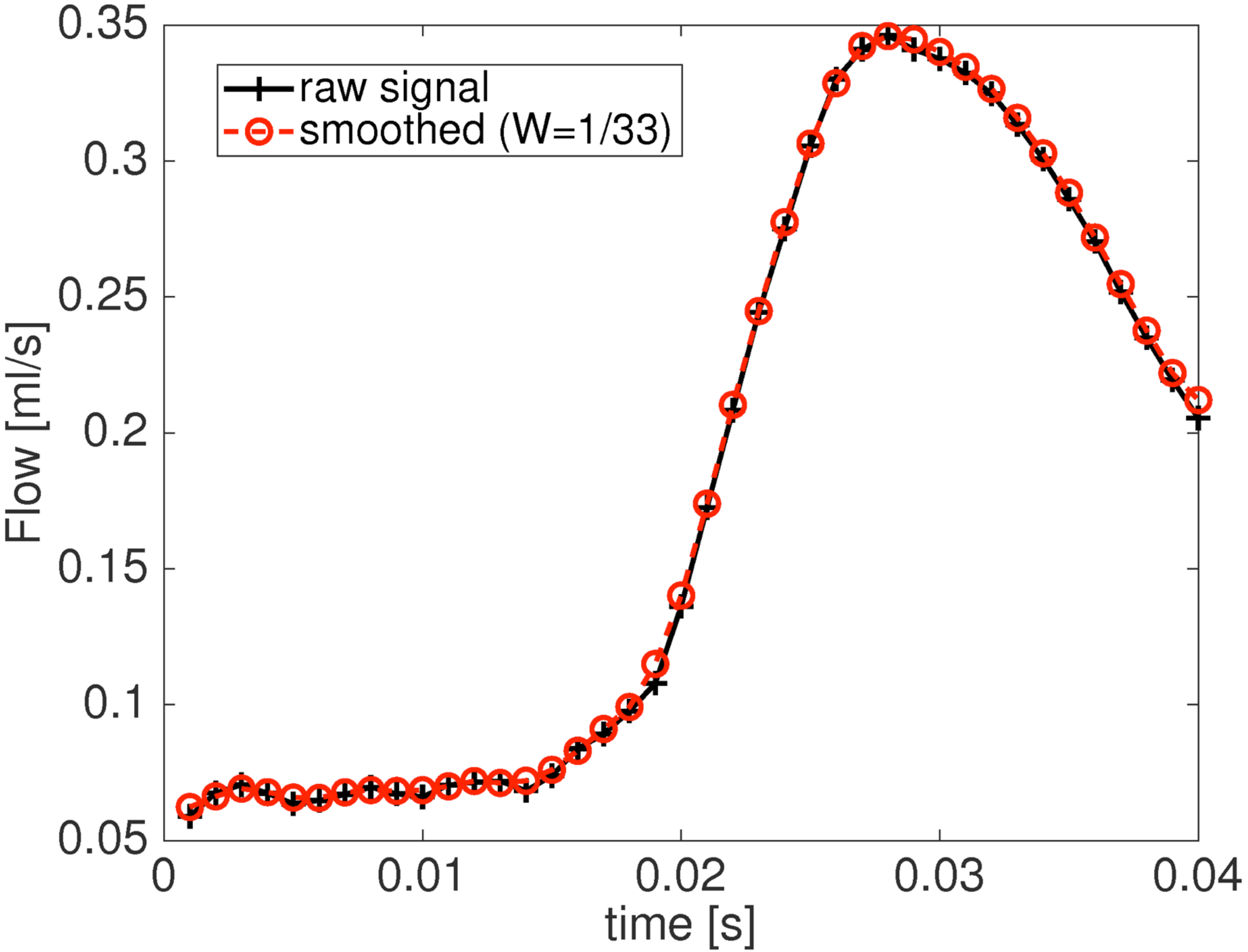

Supplement: Supplementary file 1 — Additional file 1: Figure S1–S3. Individual t-versus-x fits for the determination of PWV in order to investigate the influence of the window size (Fig. S1), the frame rates (Fig. S2) and spatial resolutions (Fig. S3). Figure S4. Magnitude responses of the temporal filtering due to the retrospective binning of projections. The finite width of the selection window acts as a moving average filter that smoothens the flow curve and suppresses higher dynamic portions. The cut-off frequencies of these low pass filters depend on the window size and are in the range between 220 Hz (W = 1/20) and 650 Hz (W = 1/60). Figure S5. Frequency analysis of a high-resolution (1 ms) flow measurement. The flow curve (left) was determined using a triggered Cartesian flow-encoding sequence. A Fourier analysis of the frequency spectrum (right) revealed only small contributions of frequency components > 300 Hz. In dashed lines: Cut-off frequency for the 1/33 selection window (approximately 350 Hz). The CMR measurement used for these plots was originally published in 2013 [21]. Figure S6. Temporal filtering with a 1/33 window. The smoothing only slightly affects the flow curve. The CMR measurement used for these plots was originally published in 2013 [21]. [file 12968_2021_725_MOESM1_ESM.pdf]
